# Intracranial Granular Cell Tumours in Three Dogs: Atypical Magnetic Resonance Imaging Features and Immunohistochemical Study

**DOI:** 10.3390/vetsci10020134

**Published:** 2023-02-09

**Authors:** Clàudia Mayor, Judit Verdés, Jaume Alomar, Rosa Novellas, Martí Pumarola, Sònia Añor

**Affiliations:** 1 Fundació Hospital Clínic Veterinari, Universitat Autònoma de Barcelona, Bellaterra, 08193 Barcelona, Spain; 2 Departament de Medicina i Cirurgia Animals, Facultat de Veterinària, Universitat Autònoma de Barcelona, Bellaterra, 08193 Barcelona, Spain; 3 Servei de Diagnòstic de Patologia Veterinària, Departament de Sanitat i Anatomia d’Animals, Facultat de Veterinària, Universitat Autònoma de Barcelona, Bellaterra, 08193 Barcelona, Spain; 4 Unitat de Patologia Murina i Comparada, Departament de Sanitat i Anatomia d’Animals, Facultat de Veterinària, Universitat Autònoma de Barcelona, Bellaterra, 08193 Barcelona, Spain

**Keywords:** granular cell tumour, magnetic resonance imaging, immunohistochemistry, brain, dog

## Abstract

**Simple Summary:**

Intracranial granular cell tumours are rare tumours of unknown cellular origin. In this case series. we describe three dogs with neurological signs that had magnetic resonance imaging of the brain, and were diagnosed with granular cell tumours through histopathological and histochemical studies. Magnetic resonance imaging characteristics were atypical, showing hypointense lesions on T2-weighted images in all cases. Immunohistochemical studies helped in characterising the tumours and excluded a Schwann cell origin. The findings of this case series indicate that granular cell tumours can be hypointense on T2-weighted magnetic resonance images, and that the cell of origin remains to be determined.

**Abstract:**

Intracranial granular cell tumours (GCT) are uncommon neoplasms of uncertain cellular origin that are rarely reported in dogs. This case series describes three aged dogs that presented with neurological signs in which magnetic resonance (MR) imaging revealed plaquelike extra-axial lesions that were hypointense on T2-weighted (T2w) images. The surgical biopsy of the lesions and necropsies were followed by histochemical characterisation with periodic acid–Schiff (PAS) staining and immunohistochemistry with ubiquitin, S-100, and SOX-10 to elucidate the cellular origin. The immunohistochemical study indicated that these intracranial GCTs were not of Schwann cell origin. In conclusion, GCTs should be considered a differential diagnosis of intracranial, extra-axial hypointense brain lesions on T2w MR images.

## 1. Introduction

Granular cell tumours (GCTs) are rare neoplasms that can be found in the nervous system of humans and dogs, and extraneurally. Intracranial GCTs were reported in several species, including rats (where they account for the most common intracranial neoplasia), dogs, one cat, a ferret, and most recently a python [1,2,3,4]. In dogs, GCTs were described as affecting the spinal cord and peripheral nerves in two cases [5,6].

The origin of these neoplasms remains uncertain: they are thought to arise from Schwann cells in people, but in other species, immunohistochemical and ultrastructural electron microscopic studies have not been able to elucidate a cell of origin. Several cells were proposed, including astrocytes, and meningeal and glial cells [2,7,8].

GCT diagnosis relies mainly upon distinctive cellular and histopathological characteristics. Typical cells are large round-to-polygonal cells with abundant eosinophilic granules within the cytoplasm and eccentric nucleus, and immunohistochemical studies revealed commonly periodic acid–Schiff (PAS) positivity [2,7,8].

Given their rarity, only 19 intracranial GCT have been reported in the literature since 1978 [2,8,9,10,11,12,13,14,15,16,17], and information regarding their typical features in advanced imaging techniques was only described in scant reports, with a total of 12 cases [8,15,16,17]. Anwer and Vernau (2013) performed the largest study of a confirmed GCT. In that study, GCTs appeared as extra-axial plaquelike lesions, hyperintense on precontrast T1-weighted (T1w) images and isohyperintense on T2-weighted (T2w) sequences. Peritumoral oedema and mass effect were also typical characteristics [16].

Here, we describe a case series of three confirmed intracranial GCT that presented to the Veterinary Teaching Hospital of the Universitat Autònoma de Barcelona from 2019 to 2022, all of which had in common an atypical magnetic resonance (MR) imaging feature: they were hypointense on T2w images. To the best of the authors’ knowledge, this is the first description of T2w hypointense intracranial GCT in dogs.

All cases underwent a complete neurological examination by a neurology and neurosurgery resident under the direct supervision of a board-certified specialist (SA). All MR images were reviewed by four counterparts: a neurology and neurosurgery resident with the board-certified specialist supervisor (SA), and by a diagnostic imaging resident (JV) with a board-certified radiologist (RN). Histopathological studies were performed by a pathology resident (JA) and board-certified pathologist (MP).

## 2. Case Presentation

### 2.1. Case 1

A 13-year-old female neutered French bulldog presented an acute onset of cluster generalised tonic–clonic seizures the same day of consultation. On presentation, general physical examination was within normal limits. Neurological examination revealed an obtunded mental status with right pleurothotonus, a tendency to circle to the right side, absent postural reactions on the left limbs and decreased on the right limbs, and absent menace response on the left eye. These abnormalities were consistent with a lesion in the right prosencephalon.

A complete blood cell count (CBC), full serum biochemical profile, thoracic radiographs, and abdominal ultrasound were all unremarkable.

**Figure 1:**
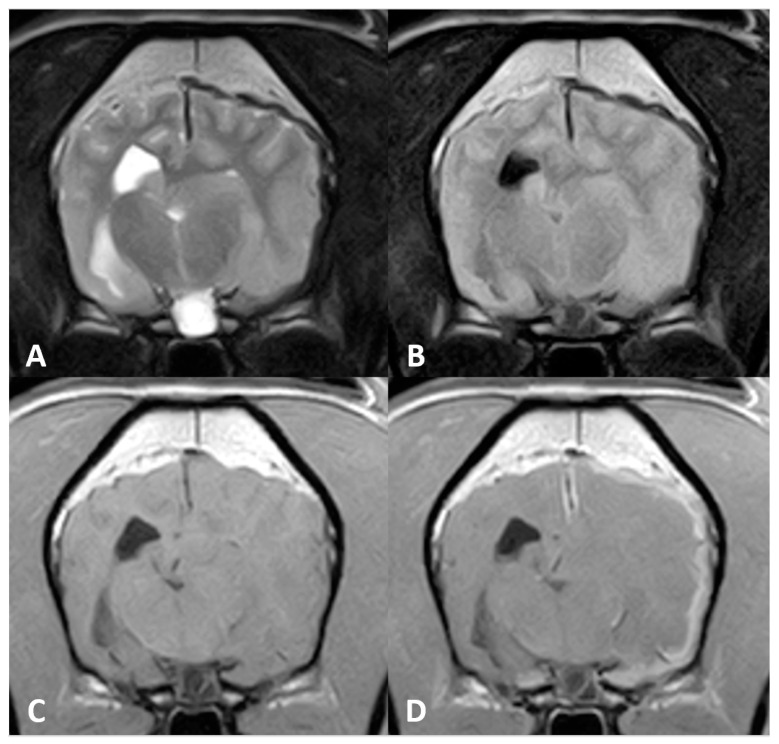
Magnetic resonance (MR) imaging of the brain of Case 1, transverse images at the level of the thalamus. A plaquelike extra-axial mass is visible covering the left hemisphere with (**A**) a marked hypointense signal on T2-weighted (T2w) sequences and (**B**) fluid attenuation inversion recovery (FLAIR). (**C**) T1-weighted (T1w) hyperintensity was not visible, but (**D**) marked enhancement after contrast administration could be detected. Note the severe mass effect and peritumoral oedema causing occlusion of the left lateral ventricle.

A routine MR imaging study was performed with T1w, T2w, fluid attenuation inversion recovery (FLAIR), postcontrast T1 (T1w+c), gradient echo (T2*) transverse sequences (see Figure 1), and T2w, T1w and T1w+c sagittal sequences.

Diffuse thickening of the meninges covering the left hemisphere was seen. A plaquelike lesion extending from the left olfactory bulb involving the meninges along the convexity of the whole hemisphere, extending to the falx cerebri and affecting partially the adjacent right cerebral hemisphere, was observed. The mass was markedly hypointense with respect to grey matter on T2w, FLAIR and T2* sequences, isointense on T1w with focal areas of hyperintensity, and showed marked and uniform enhancement after contrast administration. Adjacent to this lesion, mild diffuse T2w and FLAIR hyperintensity was present affecting the brain parenchyma. The lesion was causing a severe mass effect over the left lateral ventricle and subfalcine brain herniation of the left cerebral hemisphere. The cerebral sulci of the affected side were moderately attenuated, suggesting increased intracranial pressure.

The brain MR imaging study included the first cervical spine segments where an additional intramedullary lesion was detected: an ill-defined lesion, hyperintense on T2w images and hypointense on T1w images was present in the C2 spinal cord parenchyma, which occupied over 40% of the spinal cord diameter. No contrast enhancement was observed after gadolinium administration.

Differential diagnoses of the main lesion included primary intracranial extra-axial neoplasia (lymphoma, histiocytic sarcoma, GCT, meningioma), metastasis or an early subacute subdural haemorrhage. The C2 intramedullary lesion was consistent with syringomyelia, which could be attributed to the cerebrospinal fluid (CSF) flow disruption caused by the intracranial mass. Nonetheless, other causes of syringomyelia (such as breed-related) could not be ruled out.

A CSF sample was collected from the cerebromedullary cistern after brain MR imaging. The analysis revealed albuminocytologic dissociation: normal total nucleated cell count (TNCC) of 2 WBC/µL (reference range < 5 WBC/µL) with increased total protein concentration of 49.5 mg/dL (reference range < 20 mg/dL).

A surgical biopsy procedure through a rostrotentorial craniotomy and durectomy was performed, and a sample of the mass from the temporal meninges and a fragment of temporal bone were submitted for histopathological study.

Microscopic evaluation revealed a diffuse thickening of the dura mater with several foci of chondroid metaplasia. Neoplastic cells were large with eccentric nuclei and packed cytoplasmic granules. The adjacent temporal bone showed abundant cells of the same characteristics adhered to the periosteum, infiltrating the dura mater without displaying bone infiltration.

No immunohistochemical studies were performed even though, given the histological findings, a definitive diagnosis of a meningeal GCT was conducted, with concurrent fibrous hyperplasia and chondroid metaplasia of the dura mater.

The patient was prescribed phenobarbital at 3 mg/kg every 12 h and prednisolone 0.5 mg/kg every 12 h at tapering doses to 0.5 mg/kg every 48 h as chronic maintenance treatment. After the brain biopsy results, the patient was prescribed toceranib phosphate 2.5 mg/kg twice a week given its antitumoral and antiangiogenic effects. On routine follow-up consultations, marked improvement of clinical signs was noticed, with mild deficits in the neurological examination present in the last recheck: postural reactions were decreased in the left pelvic limb. The patient had been seizure-free for 5 months, and blood analysis revealed no adverse effects of medication. Unfortunately, the owners declined control MR imaging. One month later, the patient suffered cluster seizures and the owners elected euthanasia.

Postmortem brain examination revealed a marked diffuse enlargement of the left cerebral hemisphere causing compression over the contralateral hemisphere. Several parietal, temporal and occipital regions of the affected hemisphere were markedly attached to the meninges. Histological and immunohistochemical studies of the mass supported the antemortem diagnosis of GCT.

### 2.2. Case 2

A 13-year-old male neutered Chihuahua presented to our referral hospital for a history of cluster seizures of six-week duration, with behavioural changes and aggression during the postictal phase. On presentation, a general physical examination revealed a V/VI systolic murmur, and neurological examination did not demonstrate any abnormalities.

CBC results were within reference range, and the serum biochemistry profile showed an elevation of liver enzymes. Thoracic radiographs revealed mild cardiomegaly, and echocardiography was consistent with mitral valve degenerative disease. Abdominal ultrasonography was unremarkable.

**Figure 2:**
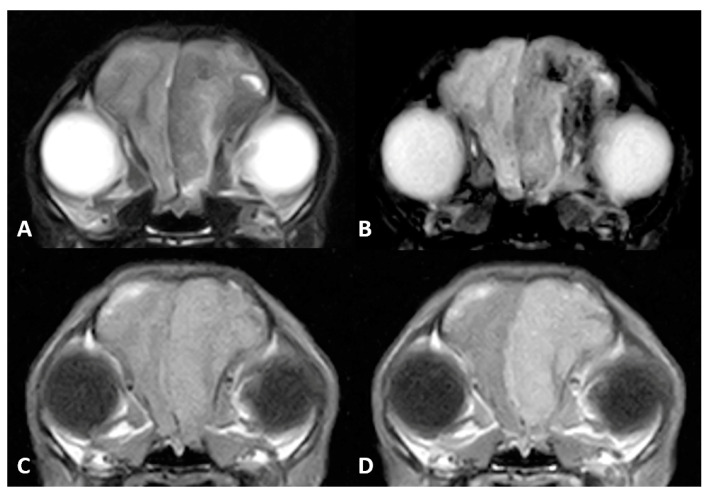
MR imaging of the brain of Case 2. (**A**) Transverse images at the level of the frontal lobes on T2w, (**B**) gradient echo sequences (T2*), (**C**) T1w and (**D**) T1w postcontrast (T1w+c). An extra-axial mass is seen in the left frontal cortex, hypointense on T2w images. There were gradient echo signal voids adjacent to the mass, compatible with intraparenchymal bleeds. Note the marked enhancement after contrast administration.

A brain MR imaging study was performed following the same brain protocol as that in Case 1 (see Figure 2), and additional transverse diffusion weighted imaging (DWI) sequences were produced. MR images revealed an extra-axial mass of ill-defined margins that extended from the left olfactory bulb throughout the left frontal and temporal regions and affected the falx cerebri. The mass was hypointense on T2w and hyperintense on T1w images, and exhibited marked homogeneous contrast enhancement. Adjacent to the mass, a second lesion could be distinguished affecting the brain parenchyma over the frontal and temporal left cortices. This lesion was hyperintense on T2w and FLAIR, with focal areas of hypointensity on T1w and several signal voids on T2* that were indicative of intraparenchymal haemorrhage surrounding the mass. A severe mass effect could be identified causing a midline shift to the right side (subfalcine herniation) and a total occlusion of the rostral part of the left lateral ventricle. Moreover, the cerebral sulci were diffusely attenuated and mild transtentorial herniation was appreciated, indicating increased intracranial pressure. No evidence of foraminal herniation was detected.

Differential diagnoses for this mass included GCT, meningioma, melanoma, histiocytic sarcoma, or metastatic tumour.

A CSF sample was collected from the cerebromedullary cistern and revealed albuminocytological dissociation: protein concentration was 72.0 mg/dL (reference < 20 mg/dL) with normal TNCC.

The patient was discharged with levetiracetam 20 mg/kg every 8 h and prednisolone 1 mg/kg every 24 h. On one month follow-up consultation the dog was seizure free and physical and neurological examinations were normal. Two months after the MR imaging, the patient’s clinical status deteriorated and it was euthanised at the owner’s request, submitting the body for a complete routinary necropsy and histopathological study.

Brain gross examination revealed a diffuse enlargement of the left cerebral hemisphere associated with an extra-axial, whitish, multilobulated mass strongly attached to the pachymeninge affecting the left olfactory bulb, left frontal area, and falx cerebri. The contralateral hemisphere showed marked compression without evidence of neoplastic infiltration.

A histopathological study showed a multinodular neoplastic proliferation, nonencapsulated with ill-defined margins, infiltrating the subjacent cortex. The tumoral cells were round with large cytoplasm containing abundant eosinophilic granules, which exhibited marked positivity after PAS staining. Even though no ultrastructural analysis was performed, the histopathologic features were highly consistent with a GCT; thus, definitive diagnosis was made.

### 2.3. Case 3

A 13-year-old female neutered Maltese presented to our referral institution in status epilepticus. The patient had suffered one isolated tonic-clonic generalised seizure the previous month, and the owners had detected lethargy and inappetence for the previous two weeks. General physical examination was normal.

Neurological examination revealed depressed mental status, ambulatory tetraparesis with marked proprioceptive ataxia of all limbs, more severe on the right side. Postural reactions were absent in both right limbs and decreased in the left pelvic limb. Cranial nerve evaluation showed absent menace response and nasal stimulation response on the right side and decreased on the left side, bilaterally decreased oculocephalic movements with mild bilateral miosis, and decreased gag reflex. These findings were consistent with multifocal brain disease, affecting the prosencephalon bilaterally (more severe on the left side) and the mesencephalon.

After emergency treatment with a single dose of diazepam 1 mg/kg IV, the seizure activity stopped, and the patient underwent diagnostic testing. CBC revealed a mild nonregenerative anaemia (PCV 36.7%, reference range 37.3–61.7%) and leucocytosis 27.73 K/µL (reference range 5.05 K/µL–16.76 K/µL) with neutrophilia 25.01 K/µL (reference range 2.95–11.64 K/µL) and mild monocytosis 1.43 K/µL (reference range 0.1–1.12 K/µL). Serum chemistry profile revealed a mild elevation of liver enzymes, azotaemia, and hyperphosphatemia.

Thoracic radiographs were unremarkable, and abdominal ultrasound findings were consistent with cholangiohepatitis and revealed the presence of a splenic thrombus.

**Figure 3:**
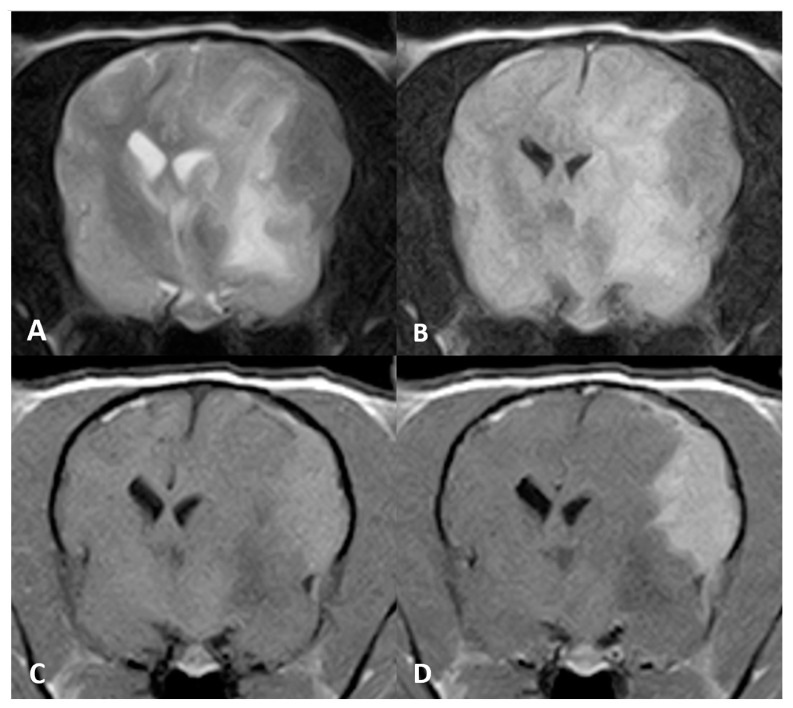
MR imaging of the brain of Case 3, transverse images at the level of the thalamus. An ovoid, plaquelike extra-axial mass is seen in the left parietal cortex. The mass was hypointense on (**A**) T2w and (**B**) FLAIR images. (**C**) Evident T1w hyperintense signal and (**D**) marked enhancement after contrast administration.

On brain MR imaging, a well-defined extra-axial mass was seen affecting the meninges covering the left hemisphere (both the parietal and temporal cortices) (see Figure 3). The mass was hypointense on T2w and FLAIR relative to grey matter and hyperintense on T1w precontrast with strong homogeneous contrast enhancement. Adjacent to the mass, T2w and FLAIR hyperintensities were evident, extending along the white matter tracts of the whole left cerebral hemisphere. The mass was causing severe mass effect with concurrent subfalcine brain herniation and mild foraminal herniation of the vermis cerebellum.

Differential diagnoses for the mass included plaquelike meningioma, histiocytic sarcoma, lymphoma and GCT.

A CSF sample was collected from the cerebromedullary cistern showing a mild pleocytosis: TNCC 6 WBCs/µL (reference range < 5 WBC/µL), and mild increase in total protein concentration 29.8 mg/dL (reference range < 20 mg/dL). Cytological examination exhibited mononuclear predominance, with marked overrepresentation of macrophages (72%) and a small lymphocyte contribution (21%).

Due to the severity of clinical signs and guarded prognosis, the owners elected euthanasia, and a complete routine necropsy and histopathological study were allowed. Gross examination showed an extra-axial plaquelike proliferative lesion highly attached to the pachymeninge, and covering the parietal and temporal regions of the left cerebral hemisphere. Multifocal areas of cerebral cortex infiltration were seen. Additionally, subfalcine brain herniation and contralateral right cerebral hemisphere compression were observed.

A microscopic examination of the mass revealed a nonencapsulated neoplastic proliferation of cells, with irregular margins that infiltrated the cerebral cortex. The neoplastic cells were large and round with abundant basophilic cytoplasm packed with granules, and they were found within a fibrovascular stroma. The histologic findings were compatible with a GCT; thus, definitive diagnosis was made.

### 2.4. Histopathological and Immunohistochemical Studies

All tissue samples from the three cases were fixed by immersion in a 10% buffered formaldehyde solution. Tissues were subsequently embedded in paraffin wax, sectioned at 3 µm, and stained with haematoxylin and eosin (HE) for routine histological assessment. Histochemical staining was performed with periodic acid–Schiff (PAS) staining. Immunohistochemical (IHC) staining was only performed on some of the properly selected sections using formalin-fixed, paraffin-embedded tissues. Primary antibodies for Ubiquitin (polyclonal rabbit antiubiquitin protein, Dako; diluted 1:400), S-100 protein (polyclonal rabbit anti-S100 protein, Dako; diluted 1:500) and SOX-10 (monoclonal rabbit anti-SOX10, Sigma; diluted 1:100) were tested. For IHC studies tissue samples were deparaffinised and incubated with endogenous peroxidase before antigen retrieval at pH 6 with 0.01 M citrate buffer.

Additionally, positive and negative controls were selected for each technique. For ubiquitin, an internal positive control was used (detecting cytoplasmic immunoreactivity within astrocytes), and for S-100 and SOX-10 detection, both a control peripheral nerve and a confirmed Schwannoma were used.

**Figure 4:**
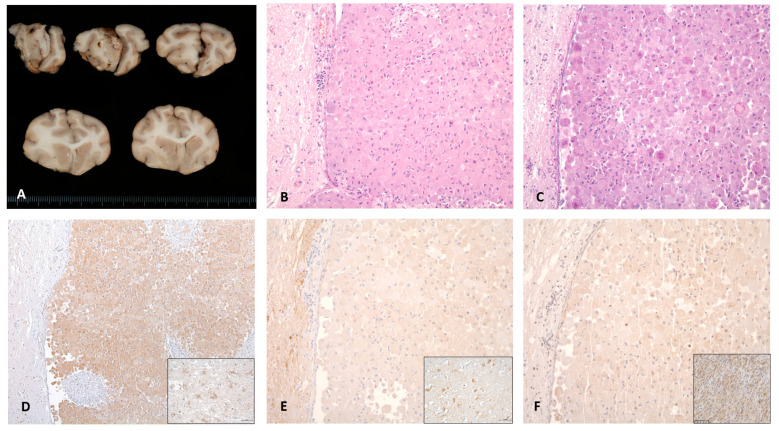
Macroscopic, histological and immunohistochemical features of Case 2. Caudal view of cerebral cross-sections. (**A**) Whitish multilobulated mass can be seen affecting the olfactory bulb with a diffuse enlargement of the left cerebral hemisphere. (**B**) Haematoxylin and eosin staining revealed neoplastic cells growing in sheets, with (**C**) a wide cytoplasm containing PAS-positive granules and (**D**) a strong expression of cytoplasmic ubiquitin protein. No immunoreactivity was detected against the (**E**) S-100 and (**F**) SOX-10 proteins. Positive controls of ubiquitin, S-100 and SOX-10 are shown in each inset.

The microscopic examination of all cases revealed densely cellular, nonencapsulated, poorly delimitated neoplastic proliferations that affected the leptomeninges and infiltrated the adjacent cortical neuropil. All tumours were mainly composed of round-to-polygonal cells arranged in sheets with variable amounts of fibrovascular stroma. The cellularity displayed abundant PAS-positive eosinophilic granules within the cytoplasm. Mild pleomorphism with low mitotic count per high power field were seen in all cases. In the adjacent neuroparenchyma, moderate spongiosis and neuroinflammation could be seen. Immunohistochemical study showed consistent results in all three cases. Tumours exhibited strong positivity for ubiquitin, variable degrees of S-100 positivity and were negative for SOX-10 immunostaining (see Figure 4).

## 3. Discussion

Intracranial GCTs are uncommon tumours in domestic animals that have scarcely been reported since their first description in dogs. To date, major investigations regarding GCTs in dogs have focused on finding a still-unknown histogenesis and defining their typical histological and ultrastructural characteristics. Nevertheless, even-though definitive diagnosis should always rely on histopathological findings, determining MR imaging characteristics could aid in reaching presumptive diagnosis of these tumours.

Only one study was published aiming to define GCT imaging features [16] that included a total of six dogs. Apart from this, 14 intracranial GCT were reported in dogs [2,9,10,11,12,13], and only 6 had a description of the imaging findings [8,15,17].

As previously stated, GCTs share several imaging features with meningiomas, which are considered the main differential diagnosis as they are the most common brain tumours in dogs. Meningiomas are extra-axial neoplasms of typical supratentorial location that most commonly affect the fronto-olfactory region [8,18,19], although they can arise from any meningeal source. They are typically round-to-ovoid tumours or they can less frequently present as plaquelike masses with a broad-based contact with the underlying bone [19,20,21]. However, plaquelike meningiomas accounted for less than 30% of cases in one study [22] and were all restricted to basilar and parasellar locations. Similarly, GCT also show a tendency to localise in the rostral cerebrum [8,16,17,19], presenting a typical plaquelike appearance, although they were also reported in sellar or parasellar locations [12].

MR tumour signal intensity can be variable for meningiomas, although they tend to be hyperintense on T2w and FLAIR, and isointense on T1w images with a strong contrast enhancement [18,21,23]. In one study including 112 confirmed meningiomas, only 2% exhibited T2w hypointensity [19,22].

Peritumoral oedema and mass effect is a typical feature of both meningiomas and GCT, and when severe, it can cause brain herniation. According to previous reports, the degree of peritumoral oedema and mass effect caused by GCT seems relatively disproportionate respective to tumour size [16,17]. In contrast, previous studies suggest that marked diffuse oedema is present in 50% of meningiomas [22,24]. In our case series, severe peritumoral oedema causing subfalcine brain herniation was detected in all cases, and a mild transtentorial brain herniation and mild foraminal brain herniation were found in cases 2 and 3, respectively. The degree of peritumoral oedema appeared subjectively disproportionate to the size of the tumours in all cases.

Another MR imaging feature suggestive of GCT is T1w precontrast hyperintensity [19,21], which is an uncommon characteristic that can be caused by different substances, including melanin (found in melanoma metastases or melanosis), lipid depositions (dermoid cysts or lipomas), calcification (necrosis), high protein content fluids or methaemoglobin (brain haemorrhages) [16,19,21]. Other primary intracranial tumours that had been described as being hyperintense on T1w images are choroid plexus tumours, ependymal tumours or gliomas [24,25,26].

Melanomas are infrequent brain tumours that contain melanin, which shortens T1 and T2 relaxation times due to its paramagnetic effect. Thus, melanocytic tumours appear hyperintense on T1w, hypointense on T2w, FLAIR and T2* sequences and typically show profound homogeneous contrast enhancement [21,27]. In humans, one primary melanocytic neoplasm is diffuse leptomeningeal melanocytosis, which typically presents as diffuse meningeal thickening [28]. Nonetheless, Anwer et al. (2013) suggested that the T1w hyperintensity of GCT was subjectively less marked than the hyperintensity observed in intracranial melanomas [16].

The vast majority of brain tumours reported in the literature are iso- to hyperintense on T2w sequences. T2w hypointensity is a rare finding that suggests presence of air-containing structures, high protein concentrations, high cellularity, mineral deposition (calcium, iron, copper) and liquids with turbulent or rapid flow [21,29]. The histopathological study of the third case described here revealed a highly dense cellular proliferation that could thereby explain the low signal intensity on T2w images. In contrast, Case 2 exhibited signs of tumoral necrosis microscopically, which could account for the characteristic signal intensities of the MR images.

In a previous review of MR imaging characteristics of brain tumours, Bentley et al. (2015) described T2w hypointensity as an uncommon finding that could be indicative of haematoma or haemorrhagic meningioma. Intracranial bleeding can present with variable signal intensities, depending on the paramagnetic effects of haemoglobin and its degradation byproducts. Early subacute haemorrhages contain methaemoglobin, which causes increased signal intensity on T1w images and hypointense signal on both T2w and T2* sequences [21,29,30]. In Case 1, one of the differential diagnoses for the MR images features observed was early subacute subdural haemorrhage, especially considering the marked hypointensity observed on T2* images.

The aim of this report was to provide a thorough description of the clinical, imaging and histopathological findings of three new confirmed GCT, considering their extremely low frequency of presentation. Consistent with previous findings, the three cases presented had prosencephalic neurologic signs.

CSF analysis was similar in all three dogs, revealing mild alterations in total protein content and a normal TNCC (Case 3 exhibited a TNCC of 6 WBCs/µL, only one cell count above reference range). In a previous study including 173 primary brain tumours, pleocytosis was found in over 60% of all cases, albuminocytologic dissociation was found in 30% of cases and only 10% of cases had normal CSF analysis. However, no GCT was included in the study [25].

GCTs were described in numerous wild and domestic species in veterinary medicine originating extracranially (lung, skin, orbit and mesentery) [2,31,32,33] and intracranially. The histogenesis of these tumours remains uncertain, although there are several hypotheses suggesting meningeal cells, histiocytes, astrocytes or neurons as potential origins in human and veterinary medicine [2]. Recently, a neural crest origin specifically from Schwann cells was postulated as the potential origin of intracranial GCTs in human medicine [34], exhibiting positive immunoreactivity to SOX-10 staining. To the authors’ knowledge (Pumarola, M.) SOX-10 is a specific immunomarker for Schwann cells and their tumours. SOX-10 was tested in canine and feline cutaneous Schwannomas and showed nuclear immunoreaction. In human medicine, Schwann cell tumours and intracranial GCTs of Schwann cell origin displayed nuclear labelling. In our three cases, granular cells showed a marked reaction to PAS staining and ubiquitin immunostaining [35], but SOX-10 immunoreactivity was not detected, and S-100 exhibited mild variable positivity [34,36]. According to these results, a neural crest and Schwann cell origin could not be confirmed.

## 4. Conclusions

Although histopathological diagnosis remains the gold standard for the definitive diagnosis of GCT, some MR imaging characteristics of these tumours are greatly distinctive and infrequent, and could be used as potential indicators. A plaquelike extra-axial mass, hyperintense on T1w, with marked homogeneous contrast enhancement and severe peritumoral oedema and mass effect, together with T2w hypointensity, could be highly suggestive of GCT. Histopathological and immunohistochemical findings of these tumours consistently reveal PAS positivity and ubiquitin immunoreactivity, although further studies should be performed in order to elucidate the cell of origin, which remains uncertain.

## Data Availability

Not applicable.
